# Monomeric C-reactive protein potentiates microglia-mediated neuroinflammation in Alzheimer’s disease *via* the PLC-PKC-MAPK-NF-κB pathway

**DOI:** 10.1016/j.jbc.2026.113527

**Published:** 2026-09-08

**Authors:** Jie Zhang, Yiyi Zhu, Qishan Zhang, Yuting Dai, Hang Liu, Jingming Shi, Yanghua Jiang, Qian Chen

**Affiliations:** 1 The Key Laboratory for High Altitude Brain Injury and Repair, School of Medicine, Xizang Minzu University, Xianyang, Shaanxi Province, China; 2 Department of Neurology, The First People’s Hospital of Chenzhou City, Chenzhou, Hunan Province, China; 3 Laboratory Medical Center of the First People’s Hospital of Chenzhou City, Chenzhou, Hunan Province, China

**Keywords:** Alzheimer's disease(AD), monomeric C-reactive protein (mCRP), neuroinflammation, microglia, PLC/PKC/MAPK/NF-κB signaling pathway

## Abstract

Neuroinflammation is a major contributor to the pathogenesis of Alzheimer’s disease (AD). Although elevated serum C-reactive protein (CRP) is associated with increased AD risk, the specific conformational forms of CRP operative in the brain and their underlying mechanisms remain unclear. Here, we combined human brain transcriptomic analysis, APP/PS1 mice studies, and functional assays in BV2 cells and primary microglia to elucidate the roles of Monomeric C-reactive protein (mCRP) in AD-related neuroinflammation. CRP was significantly upregulated in the brains of AD patients and in the hippocampus of APP/PS1 mice, where it extensively colocalized with activated microglia. mCRP directly induced M1 proinflammatory microglial polarization and synergistically enhanced neuroinflammation with amyloid-β_42_-derived diffusible ligands. Additionally, mCRP dissipated mitochondrial membrane potential and promoted intracellular and mitochondrial reactive oxygen species accumulation. Mechanistically, mCRP activated the phospholipase C–protein kinase C–mitogen-activated protein kinase–nuclear factor-κB signaling cascade, whereas the phospholipase C inhibitor U-73122 attenuated pathway activation and reduced the production of pro-inflammatory cytokines. Collectively, these findings identify mCRP as a critical mediator linking systemic inflammation to microglia-driven neuroinflammation in AD, offering new mechanistic insights into AD pathogenesis.

C-reactive protein (CRP) was synthesized by hepatocytes in response to pro-inflammatory cytokines during inflammation or infection, and was recognized as a biomarker in the serum of patients with acute inflammatory responses (1, 2). Alzheimer's disease (AD) was a neurodegenerative disorder closely associated with chronic, persistent neuroinflammation. Its pathological progression was not driven solely by amyloid β peptide (Aβ) deposition and hyperphosphorylated Tau protein forming neurofibrillary tangles; rather, on the basis of abnormal Aβ aggregation, long-term neuroinflammation further amplified neuronal damage, synaptic loss, and brain region atrophy, ultimately leading to cognitive dysfunction (3, 4). Dysregulation of peripheral immune function was frequently associated with neuroinflammation in AD (5). Therefore, whether CRP, as an inflammatory marker in serum, could serve as an indicator for neurological diseases had long attracted considerable attention. As early as more than 30 years ago, studies had demonstrated that CRP was clearly detected in senile plaques of AD patients, suggesting that the formation process of senile plaques might involve acute-phase inflammatory states and/or the production of CRP (6). Researchers demonstrated through large-scale cohort analysis (440,000 individuals) combined with neuroimaging evidence that serum CRP mediated liver-brain axis inflammation, leading to gray matter volume atrophy in the hippocampus and brainstem, as well as declines in cognition (reasoning and working memory) (7).

However, reports had shown that serum CRP levels in AD patients were lower than those in normal individuals (8, 9, 10, 11). Other studies had also suggested that elevated serum CRP levels during midlife were associated with an increased risk of AD development; however, these elevated CRP levels did not possess predictive value during the early symptomatic stage prior to clinical manifestation of AD. For a specific subgroup of patients with AD, elevated serum CRP levels remained predictive of worsening dementia severity, suggesting that AD might harbor a pro-inflammatory intrinsic characteristic (10). Elevated CRP concentrations might be strongly associated with neurodegenerative diseases and AD; however, serum CRP levels could not be simply regarded as a biomarker for AD onset, as CRP concentrations in AD patients were lower than those in patients with certain other psychiatric disorders. Nevertheless, some AD patients did exhibit elevated CRP levels, indicating the presence of inflammatory activity in this subgroup, which might render these individuals suitable candidates for anti-inflammatory treatment (12). CRP was not an indicator that reliably reflected neurodegenerative changes over time (13). In summary, previous studies had demonstrated that serum CRP could not fully serve as a marker for AD severity.

CRP was synthesized in hepatic tissue; however, it was frequently detectable in brain tissue, and elevated serum CRP levels were associated with gray matter volume in the hippocampus and brainstem regions. The underlying mechanism was that high concentrations of CRP could directly induce mitogen-activated protein kinase (MAPK) phosphorylation through FcγR (CD16/CD32), thereby activating NADPH oxidase and generating ROS that disrupted the blood-brain barrier (BBB) (14). The accumulation of mCRP within brain parenchyma appeared to be associated with vascular injury, neurodegenerative pathophysiology, and potentially AD and dementia. mCRP might undergo local tissue leakage and translocation, resulting in mCRP-positive areas filling the perivascular microenvironment in AD patients; these mCRP deposits were also co-localized with pTau and β-amyloid (15). mCRP was administered *via* intracerebroventricular injection into the brain, and a bilateral common carotid artery ligation (BCAL) model was established in CX3CR1GFP/+ mice. The results demonstrated that mCRP significantly enhanced microglial activation, elevated inflammatory response levels, accelerated neuronal loss and degenerative damage, and impaired post-ischemic behavioral performance (16). Brain injury or inflammatory neurodegenerative processes were closely associated with mCRP localization within tissue, and this protein likely played a direct role in promoting tissue damage and AD progression (15). mCRP could activate the M1 phenotype of microglia through the iNOS/NLRP3/COX-2 pathways, providing direct cellular mechanistic evidence for AD (17). These studies indicated that mCRP played an important role in AD pathogenesis by penetrating the BBB, activating microglia, and generating inflammatory pathways. Given that CRP might exert significant effects in the brains of AD patients, the question arose as to why some studies reported lower serum CRP concentrations. The underlying cause was likely associated with APOE ε4, a risk factor for AD that could suppress hepatic CRP transcription. The ε4 allele altered BBB permeability, allowing more CRP to infiltrate into the brain, thereby depleting peripheral levels; cerebrospinal fluid CRP was also reduced. Additionally, the complement system was dysregulated in the ε4 brain, and CRP, as an activator of this system, was excessively consumed (18, 19).

Although mCRP could serve as an inflammatory signal and play an important role in chronic neuroinflammation within the brain, its mechanisms of action in the brain remained to be elucidated (1). The mechanisms by which mCRP interacted with neurons and glial cells in the brain were still unclear, such as the effects of mCRP on mitochondria and intracellular ROS in the cytoplasm (2); The signaling pathway through which mCRP exerted its effects on microglia had not been identified (3); As an inflammatory molecule, how mCRP functioned during the development of inflammation in the brain, as well as its synergistic effects with Aβ, remained poorly understood, and the mechanisms by which mCRP inflammatory signals were converted into chronic neuroinflammation and oxidative stress were also unknown. Therefore, to address these questions, this study aimed to elucidate the mechanisms by which mCRP exerted its effects in the brain, reveal the processes and signaling pathways through which mCRP induced neuroinflammation and oxidative stress, and attempt to provide druggable targets.

## Results

### CRP was significantly upregulated in AD and enriched in hippocampal microglia

To explore the potential role of CRP in the pathogenesis of AD, this study collected serum samples from 65 AD patients and 30 healthy controls, all obtained from the Department of Neurology, First People’s Hospital of Chenzhou City, Hunan Province. TAD was diagnosed according to the Chinese Guidelines for the Diagnosis and Treatment of AD and the criteria established by the National Institute on Aging–Alzheimer’s Association (NIA–AA). Healthy controls were recruited from individuals undergoing routine physical examinations at the same hospital. Participants with acute infection, autoimmune disease, or recent anti-inflammatory treatment were excluded to minimize the potential effects of systemic inflammation on CRP levels. Demographic characteristics and routine biochemical parameters were collected for subsequent statistical analyses and adjustment for potential confounders (Table S1). ELISA analysis showed that serum total CRP levels did not differ significantly between patients with AD and healthy controls. In contrast, serum mCRP levels were significantly higher in patients with AD than in controls (Mann-Whitney U nonparametric rank-sum test, *p* = 0.048, ∗*p* < 0.05; Fig. 1*A*). Considering that age, gender, body mass index, liver function indicators, and lipid levels are closely related to CRP expression, Spearman correlation analysis was used to evaluate the relationship between circulating mCRP and clinical biochemical indicators. The results suggested that mCRP level was potentially correlated with serum total CRP level and blood lipid index (total cholesterol) (Fig. S1). After further adjustment for all collected clinical factors *via* multiple linear regression analysis, the diagnostic status of AD remained independently associated with elevated serum mCRP levels, whereas no significant differences related to sex or other metabolic indicators were observed (Figs. S2 and S3). Subsequently, the research team analyzed the expression pattern of CRP in human brain tissue transcriptomic data based on the GEO public database(GSE5281). Heatmap clustering analysis revealed that *CRP* expression was significantly increased in brain tissues of AD patients and showed a co-expression clustering pattern with multiple well-characterized AD-related genes, including *TREM2*, *PSEN1*, *MAPT,* and *CLU* (Fig. 1*B*), suggesting that altered CRP expression is closely associated with core molecular pathological features of AD. Differential expression analysis of the human dataset GSE48350 confirmed these findings: *CRP* expression was significantly upregulated in both the hippocampus (HIP; Wilcoxon rank-sum test, *p* = 0.0019; Fig. 1*C*) and entorhinal cortex (EC; *p* = 0.016; Fig. 1*D*). The qRT-PCR and Western blot were used to analyze the expression and distribution of CRP in brain tissues of APP/PS1 mice. The results demonstrated that both mRNA and protein levels of CRP were significantly upregulated in the hippocampus of APP/PS1 mice compared with WT mice (Fig. 1, *E* and *F*). Immunofluorescence assays showed that CRP levels in the hippocampus of APP/PS1 mice were markedly higher than those in the control group, while no significant changes were observed in the cortex (Fig. 1, *G* and *H*). These results indicated that CRP exhibits a distinct region-specific upregulation in the brain of AD mice, which is largely consistent with its expression pattern in human AD patients.

**Figure 1:**
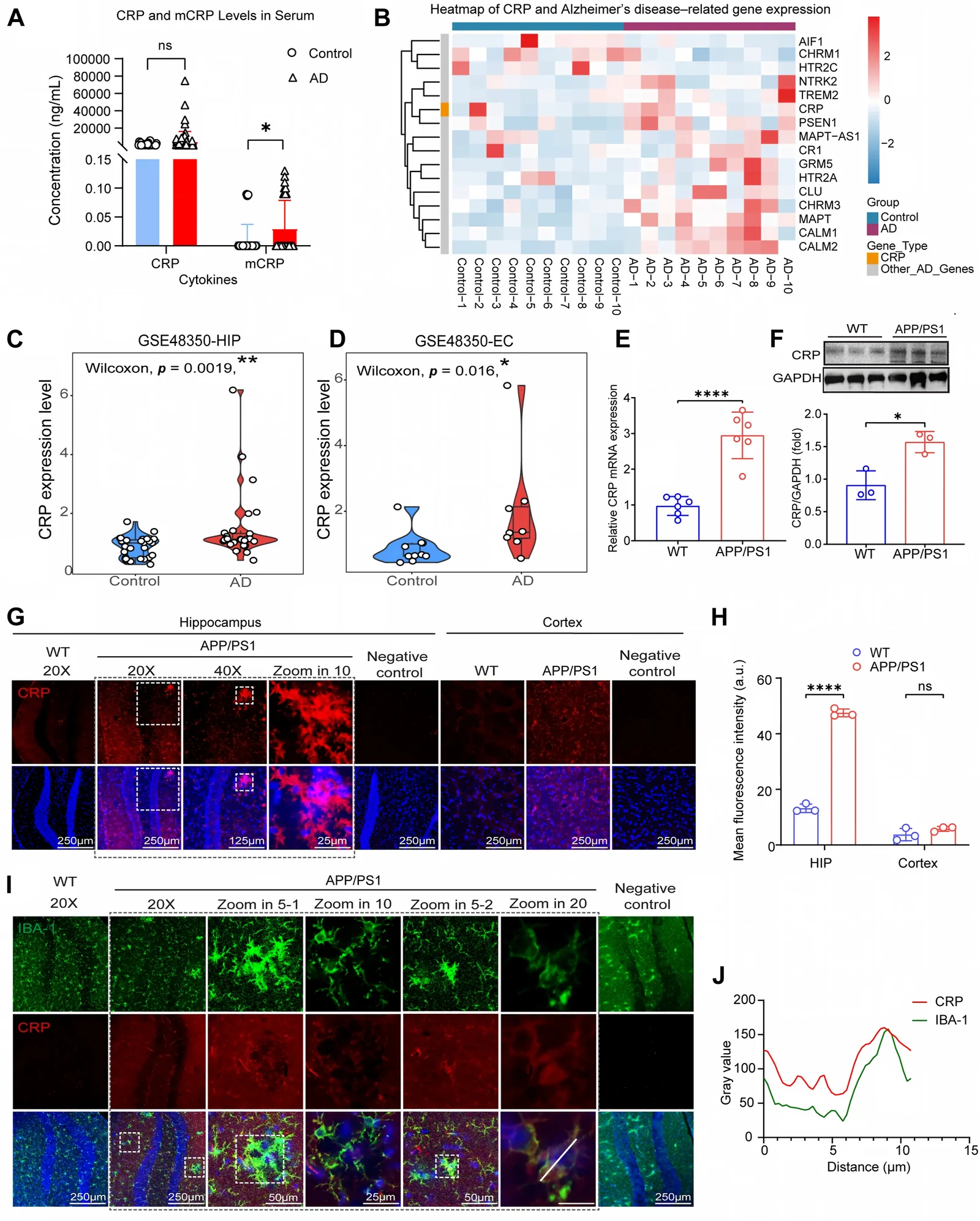
**Expression changes of CRP in AD and its spatial colocalization with microglia.***A,* serum CRP and mCRP concentrations in the healthy control group (n = 30) and AD patient group (n = 65) were detecte, the data were analyzed using the Mann-Whitney U nonparametric rank-sum test. *B,* transcriptomic data of brain tissue samples from healthy controls and AD patients were analyzed based on the GEO public database (GSE5281, n = 10 per group). Heatmaps showed the clustering distribution of CRP and multiple AD-related genes. *C* and *D, violin plots* showed the differential expression levels of CRP in the HIP and entorhinal cortex of healthy and AD patients from the GSE48350 human brain tissue transcriptome database. Wilcoxon rank sum test was used for statistical analysis. *E* and *F,* the mRNA and protein expression levels of CRP in the HIP of WT and APP/PS1 mice were quantified by RT-qPCR (n = 6) and Western blot (n = 3). Statistical analysis was performed using unpaired Student's *t* test. *G* and *H,* representative immunofluorescence images and quantitative analysis of CRP *(red)* and DAPI *(blue)* in the HIP and cortex of WT and APP/PS1 mice (n = 3). Two-way ANOVA combined with Tukey multiple comparison test was used for statistical analysis. *I and J,* representative immunofluorescence images of CRP *(red)* and IBA-1 *(green)* in brain tissues of WT and APP/PS1 mice, including low-magnification views, locally magnified views, and negative controls (*I*), as well as linear scanning analysis of the spatial distribution of fluorescence intensities for CRP and IBA-1 in colocalized regions (*J*). The scale bar represents 250 μm, 50 μm, and 25 μm, respectively. ∗*p* < 0.05, ∗∗*p* < 0.01, ∗∗∗∗*p* < 0.0001; ns, no significant difference. CRP, C-reactive protein; HIP, hippocampus; mCRP, monomeric C-reactive protein.

Microglia plays a crucial role under AD pathological conditions. Given the accumulation of CRP in the mouse brain, the research team further examined its association with microglia. Immunofluorescence double labeling and linear scanning analysis revealed colocalization of CRP with microglia in the hippocampus of APP/PS1 mice (Fig. 1, *I* and *J*). Collectively, CRP is upregulated in brain tissues of both AD patients and mouse models and colocalizes with microglia in the hippocampus.

### CRP and mCRP synergistically enhance Aβ_42_ amyloid-β42-derived diffusible ligand (ADDL)-induced inflammatory responses in microglia

The above findings demonstrated that CRP co-localizes with microglia in the hippocampus. Whether CRP and mCRP contribute to AD pathogenesis through microglia therefore remained unresolved. To address this question, *in vitro* cellular models were established using primary microglia (Fig. 2) and BV2 cells (Fig. S4), respectively. Firstly, in order to exclude the potential effect of different concentrations of CRP or mCRP on cell survival status, CCK-8 method was used to evaluate the dose safety of primary mouse microglia. The results showed that the safe concentration range could be selected within 20 μg/ml (Fig. S5). In addition, the quality of the prepared CRP was checked (Fig. S6), and a polymyxin B control group was included in key functional assays to exclude any potential endotoxin-mediated effects (Fig. S7). Next, Western blot and qRT-PCR analyses revealed that co-treatment with CRP or mCRP plus Aβ_42_ amyloid-β42-derived diffusible ligand (ADDLs) increased expression of the proinflammatory cytokines IL-6, IL-1β, and TNF-α to a greater extent than either treatment alone, exhibiting prominent synergistic potentiating effects. The interaction index (II), calculated as *II = (R*_*combined*_ *- R*_*control*_*)/(R*_*CRP/mCRP*_ *+ R*_*Aβ42-ADDLs*_ *- 2R*_*control*_*)*, exceeded one for all cytokines examined, where *R*_*combined*_ refers to the measured response value for the group co-treated with CRP/mCRP and Aβ_42_-ADDLs; *R*_*CRP/mCRP*_ and *R*_*Aβ42-ADDLs*_ represent the response values of the CRP/mCRP single-treatment group and the Aβ_42_-ADDLs single-treatment group, respectively; and *R*_*control*_ denotes the response value of the control group. Although the two cell models differed in sensitivity and activation threshold, both demonstrated that CRP and mCRP synergistically amplified Aβ_42_ ADDL-triggered inflammatory responses at the mRNA and protein levels. These data support a model in which CRP and mCRP function as amplifiers of microglial inflammation.

**Figure 2:**
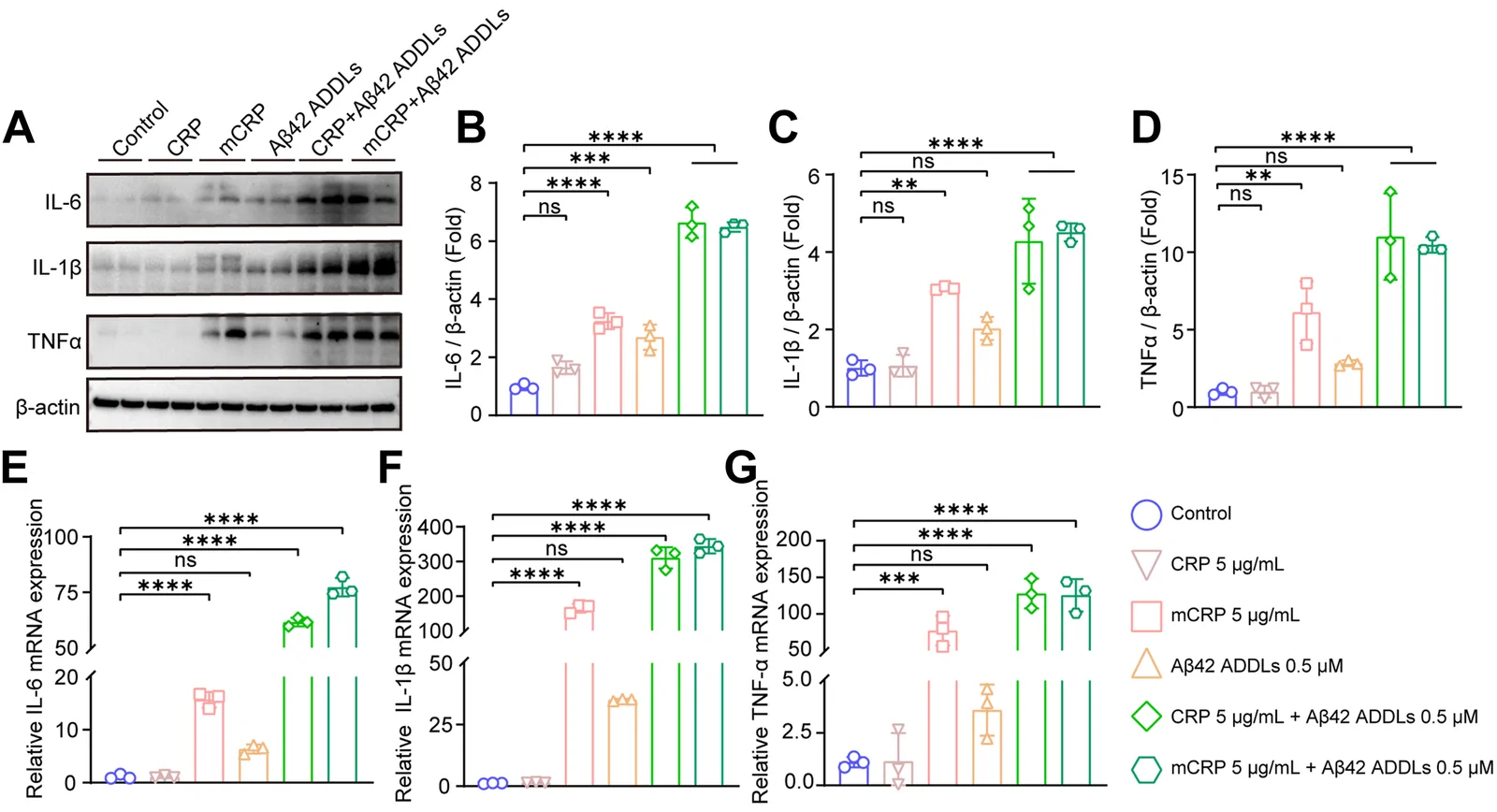
**CRP and mCRP synergistically amplify the inflammatory response of microglia with Aβ_42_ ADDLs.** Primary mouse microglia were treated under different conditions for 12 h, including the control group, CRP alone group (5 μg/ml), mCRP alone group (5 μg/ml), Aβ_42_ ADDLs alone group (0.5 μM), CRP + Aβ_42_ ADDLs combined treatment group, and mCRP + Aβ_42_ ADDLs combined treatment group. *A–D,* western blot was performed to detect the protein expression levels of inflammatory factors IL-6, IL-1β, and TNF-α in primary mouse microglia under the above different treatments, with β-actin as the internal reference, followed by quantitative analysis of protein expression in each group (n = 3). *E–G,* qRT-PCR was conducted to detect the relative mRNA expression levels of *Il6*, *Il1b*, and *Tnfα* under different treatments (n = 3). All quantitative data are expressed as mean ± SD, and statistical analysis was performed as described in the Materials and Methods section. ∗∗*p* < 0.01, ∗∗∗*p* < 0.001, ∗∗∗∗*p* < 0.0001; ns, no significant difference. ADDL, amyloid-β42-derived diffusible ligand; mCRP, monomeric C-reactive protein.

### mCRP induced the M1 phenotype in primary microglia

Increased secretion of IL-6, IL-1β, and TNF-α was closely associated with the M1 phenotype of microglia (20, 21). In AD, since both CRP and mCRP were involved in the inflammatory response of microglia and led to elevated expression of inflammatory factors, CRP and mCRP were highly likely to participate in inducing the M1 phenotype in microglia. However, it remained unclear which form of CRP or mCRP exerted a stronger inductive effect on microglia. Subsequently, the primary mouse microglia were co-incubated with CRP or mCRP at different concentrations (5, 10, 20 μg/ml) for 12 h qRT-PCR results showed that mCRP significantly upregulated the expression of proinflammatory genes (*Il6*, *Tnfα*, *Il1b*, and *Cox2*) in a dose-dependent manner, among which the induction magnitude in the 20 μg/ml mCRP treatment group reached 40 to 130 folds that of the blank control group. In contrast, the inductive effect of native pentameric CRP was significantly weaker or not significant under the same conditions (Fig. 3, *A*–*D*). Further analysis confirmed the regulatory effect of mCRP on M1 phenotypic polarization of microglia. mCRP induced a stronger upregulation of CD68 expression in microglia than CRP (Fig. 3*E*). Meanwhile, CRP had no obvious effect on CD206 expression compared with mCRP (Fig. 3*F*). In addition, co-incubation of mCRP with microglia resulted in markedly enhanced CD86 expression, which was consistently verified by immunofluorescence and flow cytometry (Fig. 3, *G* and *H*). Transcriptional level analysis further revealed that mCRP downregulated *Mrc1* and upregulated *Cd86* mRNA expression (Fig. 3, *I* and *J*). Collectively, mCRP exhibited a stronger ability than CRP to promote the transformation of microglia toward the M1 phenotype.

**Figure 3:**
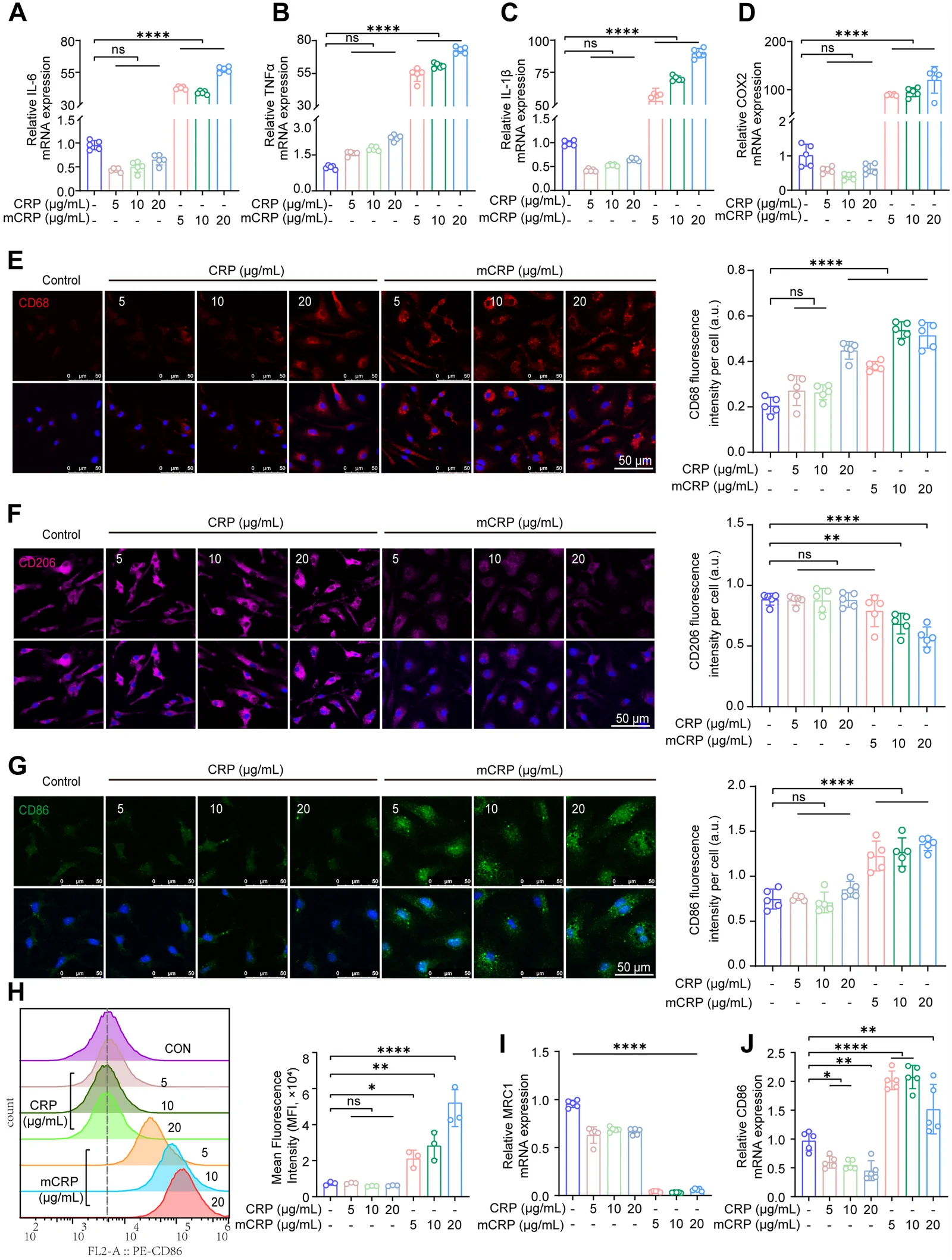
**Regulatory effects of different concentrations of CRP and mCRP on the inflammatory phenotype and polarization status of primary microglia.** Primary microglia were coincubated with native CRP or mCRP at different concentrations (5, 10, and 20 μg/ml) for 12 h. *A–D,* qRT-PCR was used to detect mRNA expression levels of proinflammatory-related genes in primary microglia, including *Il6*, *Tnfα*, *Il1b*, and *Cox2* (n = 5). *E–G,* immunofluorescence staining was performed to detect representative images and quantitative results of fluorescence intensity of CD68 (*red*, *panel E*), CD206 (*magenta*, *panel F*), and CD86 (*green, panel G*) in primary microglia after intervention. Nuclei were stained with DAPI *(blue)*. The scale bar represents = 50 μm (n = 5). *H,* flow cytometry was used to detect CD86 expression levels in primary microglia. Representative histograms are shown on the *left*, and quantitative analysis of mean fluorescence intensity is shown on the right (n = 3). *I* and *J,* qRT-PCR was used to detect mRNA expression levels of *Mrc1* (*I*) and *Cd86* (*J*) under different treatment conditions (n = 5). Data were presented as mean ± SD. Statistical analysis was performed using one-way ANOVA followed by *post hoc* multiple comparisons test. ∗*p* < 0.05, ∗∗*p* < 0.01, ∗∗∗∗*p* < 0.0001; ns, no significant difference. CRP, C-reactive protein; mCRP, monomeric C-reactive protein.

### mCRP mediated neuroinflammation in primary microglia by inducing mitochondrial dysfunction

Our previous results showed that mCRP significantly induced M1 polarization in primary microglia with massive production of inflammatory factors, whose generation is often accompanied by alterations in mitochondrial function (22). Therefore, mCRP was highly likely to induce changes in mitochondrial function. Accordingly, primary microglia were co-incubated with CRP or mCRP for 12 h, and mitochondrial membrane potential (ΔΨm) was evaluated using JC-1 fluorescent probe staining. The results showed that mCRP treatment triggered a marked shift of JC-1 fluorescence from red J-aggregates (indicating normal membrane potential) to green J-monomers (indicating depolarization), and the red/green fluorescence ratio decreased in a dose-dependent manner, signifying obvious mitochondrial depolarization in primary microglia. In contrast, CRP at the same concentration only induced slight changes (Fig. 4, *A*–*D*). Using the DCF-DA probe, quantitative analyses by microplate reader and immunofluorescence consistently revealed that mCRP significantly increased intracellular total reactive oxygen species (ROS) production. The DCF-DA fluorescence intensity in the 20 μg/ml mCRP-treated group was approximately 3-fold higher than that in the control group (Fig. 4, *E*–*G*). Mitochondrial superoxide anion was further specifically detected using MitoSOX Red probe, and the results confirmed that mCRP treatment markedly elevated mitochondrial ROS levels (Fig. 4, *H* and *I*), suggesting that mitochondria are the main source of oxidative stress induced by mCRP in microglia. Taken together, these findings demonstrated that mCRP caused mitochondrial membrane potential loss and increased ROS production.

**Figure 4:**
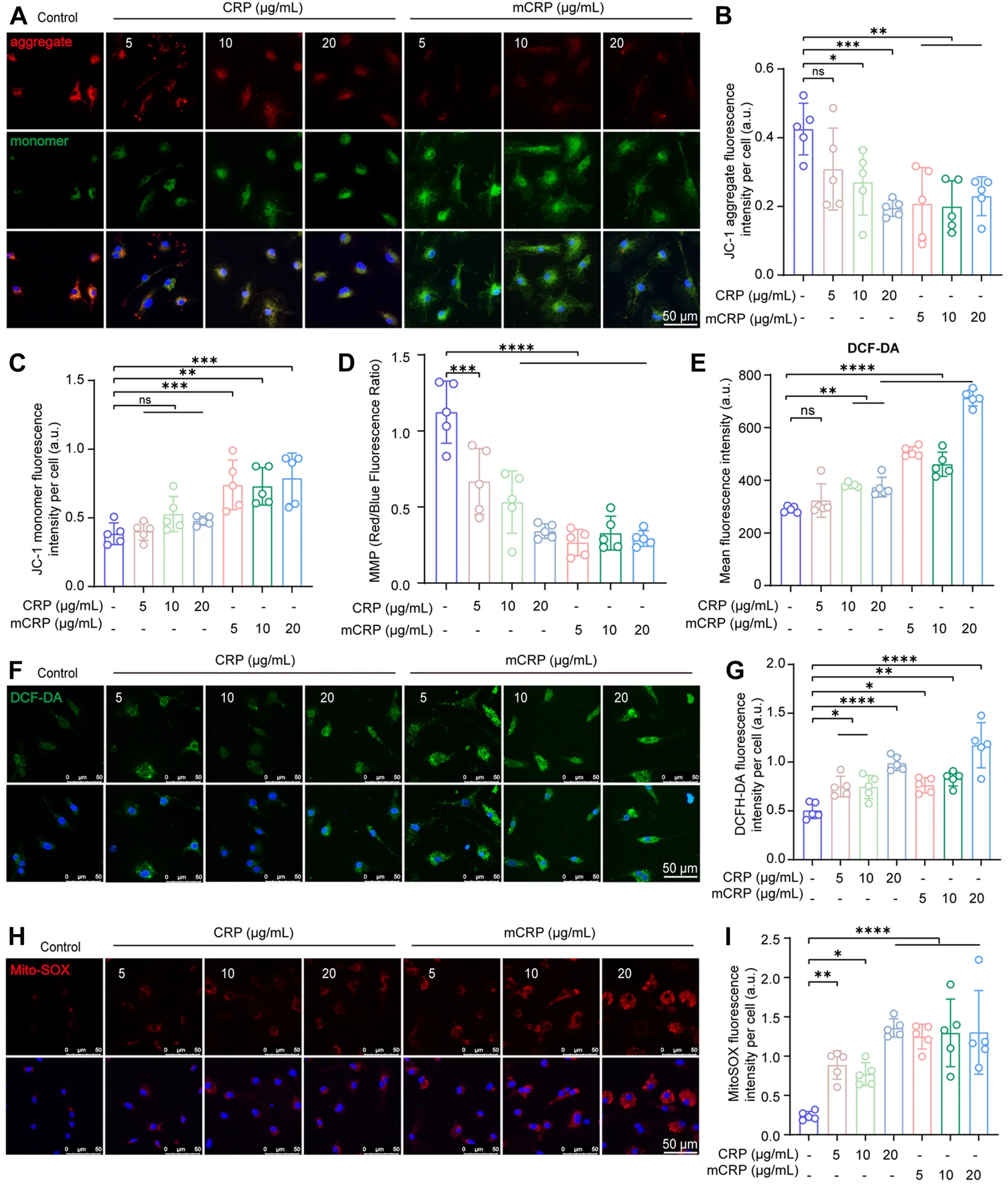
**Effects of CRP and mCRP on mitochondrial function and oxidative stress in primary microglia.** Primary microglia were co-incubated with CRP and mCRP at different concentrations (5, 10, and 20 μg/ml) for 12 h. *A–D,* representative fluorescence images and quantitative analysis of the mean fluorescence intensity of mitochondrial membrane potential (ΔΨm) detected by immunofluorescence staining based on the JC-1 probe. *Red* fluorescence indicated JC-1 aggregates (high ΔΨm), *green* fluorescence indicated JC-1 monomers (low ΔΨm), and nuclei were counterstained with DAPI *(blue)*. MMP (ΔΨm) was expressed as the JC-1 *red/green* fluorescence ratio. The scale bar represents = 50 μm (n = 5). *E–G,* intracellular reactive oxygen species (ROS) levels in each group were evaluated by measuring DCF-DA expression using a microplate reader and immunofluorescence staining, respectively. The scale bar represents = 50 μm (n = 5). *H* and *I,* representative fluorescence images and quantitative analysis of mitochondrial superoxide anion production in primary microglia detected by MitoSOX Red staining. Nuclei were counterstained with DAPI *(blue)*. The scale bar represents = 50 μm (n = 5). Data were presented as mean ± SD from at least three independent experiments. Statistical analysis was performed using one-way ANOVA followed by *post hoc* multiple comparisons test. ∗*p* < 0.05, ∗∗*p* < 0.01, ∗∗∗*p* < 0.001, ∗∗∗∗*p* < 0.0001; ns, no significant difference. CRP, C-reactive protein; mCRP, monomeric C-reactive protein.

### mCRP mediated inflammatory activation of microglia through the phospholipase C-protein kinase C - mitogen-activated protein kinase-nuclear factor-κB signaling axis

To elucidate the signaling mechanism by which mCRP participates in microglial neuroinflammation, GEO transcriptomic data (GSE5281) were further analyzed. A total of 2534 genes significantly positively correlated with *CRP* (Spearman r > 0.3, *p* < 0.05) were screened from differentially expressed genes in brain tissue samples of AD patients and healthy controls. KEGG pathway enrichment analysis revealed that the top 10 classic signaling pathways in which these differentially expressed genes were significantly enriched mainly included the PI3K-Akt, MAPK, nuclear factor-κB (NF-κB), TNF, IL-17, phosphatidylinositol signaling system, Rap1/Ras, TRP channel-mediated inflammatory mediator regulation, and focal adhesion pathways (Fig. 5*A*). Most of these pathways were closely related to inflammatory responses, signal transduction, and immune activation, suggesting that mCRP is widely involved in neuroinflammation during AD pathogenesis. Further literature survey and analysis indicated that the phosphatidylinositol signaling system is highly associated with the function of the phospholipase C (PLC) family (23, 24). Coexpression network analysis showed that CRP was significantly co-expressed with multiple PLC pathway genes (*PLCB1*, *PLCB2*, *PLCB4*, *PLCD3*, *PLCE1*, *PLCG1*, *PLCG2*) in brain samples from AD patients, whereas such correlation was absent in the healthy control group (Fig. 5*B*), suggesting a potential functional interaction between CRP and the PLC signaling pathway under AD pathological conditions. Based on the above bioinformatic clues, the PLC- protein kinase C (PKC)-MAPK-NF-κB signaling axis in primary microglial models was further investigated. Western blot results showed that mCRP treatment significantly upregulated the phosphorylation levels of MAPK family members ERK1/2, JNK, and p38 (increased by 2.06-fold, 2.80-fold, and 4.47-fold, respectively) and elevated total protein expression of PKC. The phosphorylation level of the NF-κB p65 subunit was also significantly increased. Pharmacological intervention using the specific PLC inhibitor U-73122 (10 μM) revealed that U-73122 pretreatment markedly attenuated mCRP-induced phosphorylation of ERK1/2, JNK, p38, and p65 (Fig. 5, *C*–*G*), indicating that PLC serves as a key upstream node in this signaling cascade. mCRP treatment promoted the translocation of NF-κB p65 from the cytoplasm to the nucleus, with a significantly increased nuclear/cytoplasmic fluorescence ratio (approximately 1.57-fold increase), and this effect was also significantly suppressed by U-73122, while total p65 expression showed no obvious change (Fig. 5, *H*–*J*), suggesting that PLC functions by regulating the phosphorylation and nuclear translocation of NF-κB rather than its total protein level. Consistent with the regulation of upstream signaling pathways, at the downstream functional level, U-73122 pretreatment effectively blocked the marked elevation of pro-inflammatory cytokines IL-1β, IL-6, and TNF-α at both transcriptional and protein levels induced by mCRP (Fig. 5, *K*–*M*).

**Figure 5:**
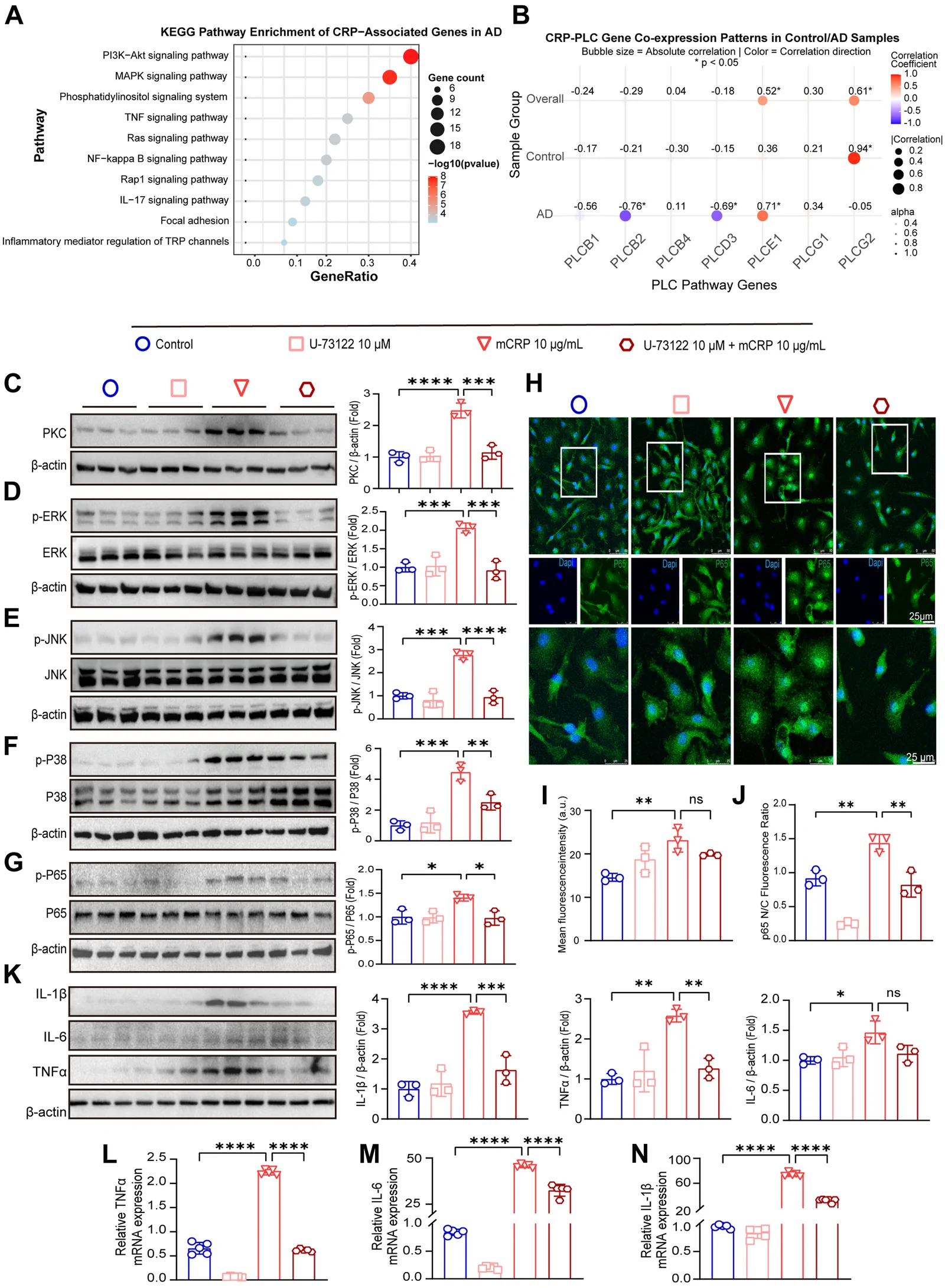
**mCRP activates the PLC–PKC–MAPK/NF-κB signaling pathway and induces proinflammatory cytokine expression in microglia.***A,* KEGG pathway enrichment analysis was performed on differentially expressed genes between healthy control and AD patient samples based on the GEO database (GSE5281). Bubble size represented gene count, and color represented −log10(*p* value). *B,* correlation analysis between CRP and PLC pathway-related genes among differentially expressed genes in healthy control and AD patient samples. Bubble size represented the absolute value of the correlation coefficient, and color represented the direction and strength of the correlation. *C*–*G,* representative Western blot bands and quantitative analysis of key proteins in primary microglia under different treatment conditions: control group, U-73122 (10 μM), mCRP (10 μg/ml), and U-73122 + mCRP. Proteins included PKC (*C*), p-ERK/ERK (*D*), p-JNK/JNK (*E*), p-p38/p38 (*F*), and p-p65/p65 (*G*). β-actin was used as the internal reference of PKC protein (n = 3). *H*–*J,* representative immunofluorescence staining images and magnified views of p65 *(green)* and nuclei (DAPI, *blue*) in primary microglia (the scale bar = 25 μm). Quantitative results were expressed as mean fluorescence intensity of p65 and its nuclear/cytoplasmic ratio, reflecting NF-κB activation and nuclear translocation. *K,* western blot detection and quantitative analysis of proinflammatory cytokines IL-1β, IL-6, and TNF-α (n = 3). *L–N,* relative mRNA expression levels of inflammatory factors *Tnfα*, *Il6*, and *Il1b* (n = 5). Data were presented as mean ± SD. Statistical analysis was performed using one-way ANOVA followed by Tukey’s *post hoc* multiple comparisons test. Significance was defined as follows: ns, no significant difference; ∗*p* < 0.05, ∗∗∗*p* < 0.001, ∗∗∗∗*p* < 0.0001. Statistical comparisons included the mCRP-treated group *versus* the control group, and the mCRP + PLC inhibitor group *versus* the mCRP group. CRP, C-reactive protein; mCRP, monomeric C-reactive protein.

### mCRP activated PLC-dependent neuroinflammation in APP/PS1 mice

Previous results have confirmed that mCRP mediates the inflammatory activation of microglia through PLC-PKC-MAPK-NF-κB signaling axis. Next, to determine whether this mechanism contributes to AD pathogenesis *in vivo*, 5-month-old male WT and APP/PS1 mice were treated by intraperitoneal injection of exogenous mCRP (200 μg/kg) with or without the PLC inhibitor U-73122 (1 mg/kg) (Fig. 6, *A* and *B*). CRP expression was higher in the hippocampus of APP/PS1 mice than in WT mice, and mCRP treatment further increased CRP accumulation in the brain (Fig. 6, *C* and *D*). Consistent with the *in vitro* findings, mCRP treatment activated PLC downstream signaling in APP/PS1 mice: PKC expression was up-regulated, and phosphorylation of ERK, JNK, p38 MAPK, and NF-κB p65 was increased (Fig. 6, *E*–*I*). In addition, mCRP treatment promoted neuroinflammation in the brains of AD mice, as shown by increased protein and mRNA levels of IL-1β, IL-6, and TNF-α (Fig. 6, *J*–*M*); Conversely, U-73122 attenuated mCRP-induced MAPK/NF-κB signaling activation and reduced pro-inflammatory cytokine expression. Together, these *in vivo* data confirm that mCRP promoted neuroinflammation in APP/PS1 mice by activating the PLC/PKC/MAPK/NF-κB signaling axis, and that PLC blockade alleviates mCRP-mediated neuroinflammation.

**Figure 6:**
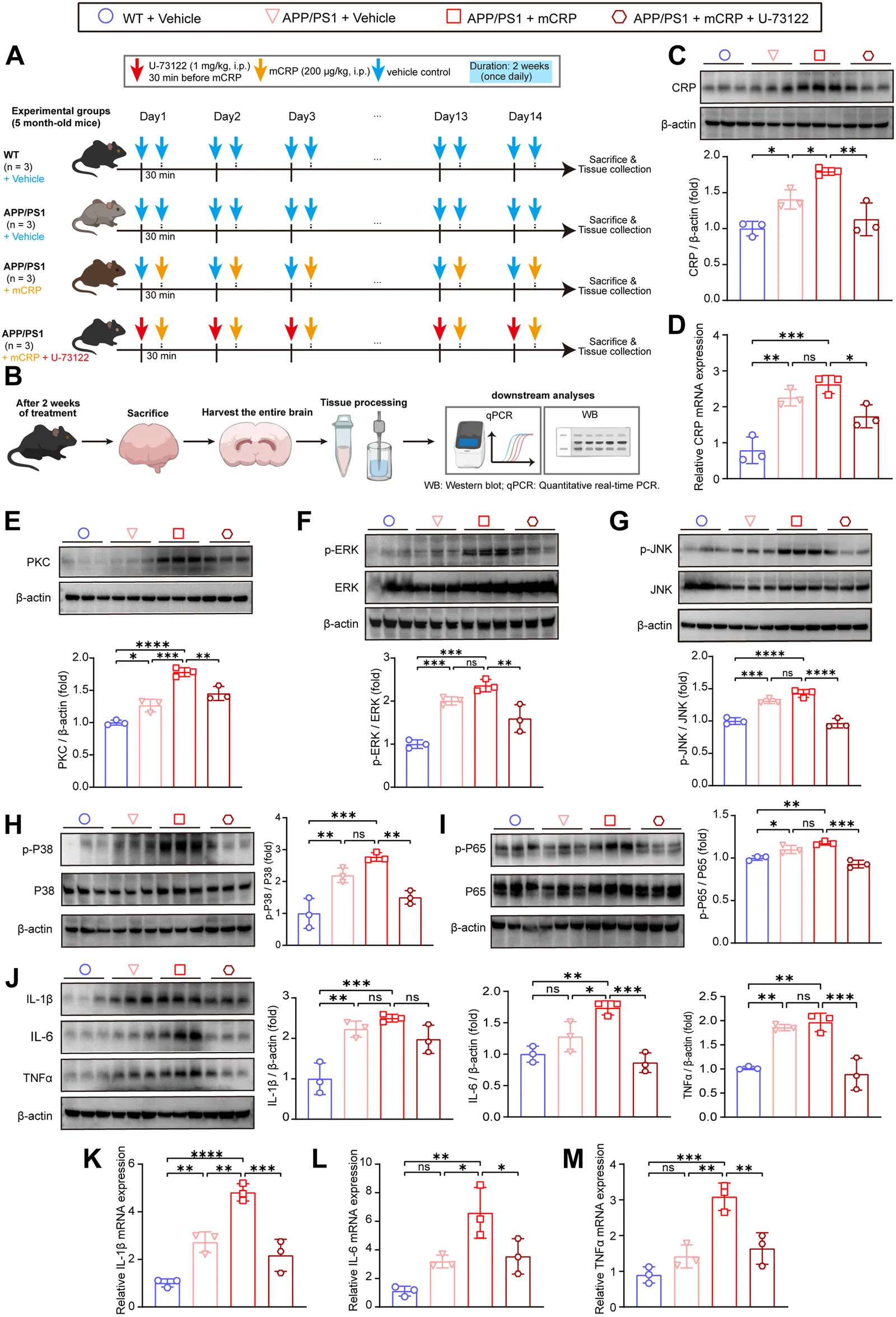
**mCRP promotes the neuroinflammatory response in APP/PS1 mice by activating the PLC/MAPK/NFκB signaling pathway.***A,* schematic design of the animal experiment. 5-month-old male WT mice and APP/PS1 transgenic mice were divided into four groups: WT + Vehicle group, APP/PS1 + Vehicle group, APP/PS1 + mCRP group and APP/PS1 + mCRP + U-73122 group (n = 3). The mice were intraperitoneally injected with Vehicle (control treatment), mCRP (200 μg/kg) and/or PLC inhibitor U-73122 (1 mg/kg) for 2 weeks *B,* schematic diagram of the experimental process. *C and D,* western blot and qRT-PCR detected the expression levels of CRP protein and mRNA in brain tissue of mice in different treatment groups. *E–I,* the expression levels of PKC, total and phosphorylated ERK, JNK, p38 MAPK and NF-κB p65 were detected by Western blot. *J–M,* western blot and qRT-PCR detected the expression levels of inflammation factors (IL-1β、IL6 and Tnfα) protein and mRNA in brain tissue of mice in different treatment groups. The comparison between groups was conducted using one-way ANOVA, followed by Tukey's multiple comparison test for statistical analysis. ∗*p* < 0.05, ∗∗*p* < 0.01, ∗∗∗*p* < 0.001, ∗∗∗∗*p* < 0.0001; ns, no significant difference. CRP, C-reactive protein; mCRP, monomeric C-reactive protein.

### Concluding summary

In summary, these results demonstrate that mCRP induces a pro-inflammatory phenotype in microglia through activation of the PLC-dependent PKC-MAPK-NF-κB signaling pathway in AD. Pharmacological blockade of PLC effectively attenuates this inflammatory response, laying a theoretical foundation for targeted intervention in mCRP-mediated neuroinflammation.

## Discussion

Neuroinflammation was recognized as a major cause of AD pathogenesis (25). CRP was an acute-phase reactant protein synthesized in the liver, primarily used to evaluate systemic inflammatory status. Epidemiological studies had confirmed a significant association between elevated circulating CRP levels and an increased risk of AD (26, 27). However, whether it was directly involved in the pathological process of AD had long been controversial.

In the present study, using clinical data combined with human brain transcriptome database analysis and the APP/PS1 mouse model, we found that CRP levels were upregulated in the brain tissue of AD patients and in the hippocampus of model mice, and showed a high spatial colocalization with activated microglia (Fig. 1), suggesting that CRP might influence the function of key brain regions. CRP exists in two conformational forms *in vivo*: the native pentameric form and the depolymerized monomeric form. Previous studies indicated that CRP irreversibly depolymerized into mCRP under conditions of tissue injury, on the surface of activated cell membranes, or in biomembrane microenvironments, and the latter exhibited significantly enhanced proinflammatory activity (28, 29). In the pathological context of AD, multiple conditions favorable for the conversion of CRP to mCRP existed in the brain, including (1): the lipid microenvironment on the surface of Aβ plaques could promote CRP depolymerization (30). (2)Membrane phospholipids released by activated microglia and damaged neurons provided a catalytic platform for depolymerization (3); Decreased pH and oxidative stress in the AD brain further accelerated this conformational transition. Therefore, in the microenvironment where microglia and Aβ plaques coexisted, CRP could be continuously converted into mCRP with strong proinflammatory activity. This finding also echoed recent research progress on local CRP production. For instance, in addition to the liver, several studies had confirmed that neurons, microglia, and astrocytes could locally synthesize CRP under inflammatory stimulation (31). In addition, dysfunction of the BBB was one of the early pathological features of AD (32). Peripheral CRP could enter the brain parenchyma through the damaged BBB, while the inflammatory microenvironment in the brain could stimulate glial cells to synthesize CRP locally. Both events jointly maintained and enhanced high-level CRP expression in the brain. Therefore, the role of CRP in the AD brain might far exceed its traditionally recognized function as an inflammatory marker, but instead acted as a central hub molecule linking systemic peripheral inflammation with central neuroinflammation.

During AD pathogenesis, persistent stimulation by Aβ oligomers shifted microglia toward the M1 proinflammatory phenotype, leading to the release of large amounts of proinflammatory factors such as TNF-α, IL-1β, and IL-6, as well as ROS, thereby exacerbating neuronal damage (33). The present study found that mCRP directly induced polarization of primary microglia toward the M1 proinflammatory phenotype and upregulated the expression of classic M1 markers including TNF-α, IL-1β, and IL-6 (Fig. 3). Similarly, the same treatment was performed in the immortalized BV-2 microglia cell line. Although the results were not identical due to differences in cell type sensitivity and activation threshold, a consistent trend was observed. More importantly, combined treatment with mCRP and Aβ_42_ oligomers (ADDLs) exerted a marked synergistic enhancing effect (Fig. 2). In the brains of AD patients, Aβ activated microglia *via* multiple receptors such as TLR2/4, RAGE, and CD36 (34, 35, 36). The present study indicated that mCRP further amplified the inflammatory response through the PLC/PKC/MAPK/NF-κB pathway (Figs. 5 and 6), which was independent yet might crosstalk with the Aβ signaling cascade. The synergistic effect between the two could be attributed to several aspects (1): mCRP and Aβ might activate distinct upstream receptors respectively, but eventually converged on NF-κB as a key transcription factor node, resulting in signal integration (2); PKC activation induced by mCRP could enhance the expression or sensitivity of Aβ receptors on the cell membrane, lowering the response threshold of microglia to Aβ stimulation (3); Mitochondrial dysfunction and ROS accumulation caused by mCRP might further activate the NLRP3 inflammasome, which had been identified as a central effector in Aβ-mediated neuroinflammation (37). Such multi-level signal synergy and amplification provided a reasonable explanation for the disproportionately intense inflammatory response observed in the brains of AD patients. In addition, the mCRP-Aβ synergy observed in this study might also account for a long-standing clinical paradox: elderly individuals with systemic inflammation or metabolic syndrome (characterized by high CRP levels) exhibited a significantly increased risk of AD onset and accelerated disease progression (38).

Another important finding of the present study was that mCRP significantly induced a decrease in mitochondrial membrane potential and massive accumulation of intracellular and mitochondrial ROS in microglia (Fig. 4). Mitochondrial dysfunction was one of the core pathological features of AD (39). The reduced mitochondrial membrane potential induced by mCRP supported and sustained the M1 proinflammatory phenotype at the metabolic level. Damaged mitochondria were not only the main source of ROS but also released damage-associated molecular patterns such as mitochondrial DNA and cardiolipin, which in turn activated innate immune signaling pathways including cGAS-STING and NLRP3 (40). This formed a vicious cycle of mitochondrial damage, inflammatory activation, and further mitochondrial injury. Meanwhile, ROS accumulation promoted the degradation of IκBα through oxidative modification, further enhancing the nuclear translocation and transcriptional activity of NF-κB (41). As the initiating node of this signaling cascade, the present study demonstrated that its specific inhibitor U-73122 effectively blocked downstream PKC activation, phosphorylation of MAPKs (ERK, JNK, p38), NF-κB nuclear translocation, and proinflammatory cytokine production. It is confirmed that PLC plays a central role in CRP-mediated signaling in neuroinflammation both *in vitro* and *in vivo* AD models (Figs. 5 and 6).

Although this study provided a relatively comprehensive chain of evidence, it still had several limitations that needed to be addressed in subsequent research. While the present study clarified the PLC/PKC/MAPK/NF-κB signaling cascade, the upstream specific receptor of mCRP had not been fully identified. Subsequent studies could systematically screen for the functional receptor of mCRP on microglia using single-cell RNA sequencing combined with receptor knockout or blockade strategies, which represented an important direction for future research. Meanwhile, attention should also be paid to the spatiotemporal dynamics of mCRP in the AD brain, including changes in its concentration at different disease stages, differences in its distribution across various brain regions, and its relative accumulation in the perivascular space and brain parenchyma.

In summary, this study systematically revealed that mCRP was involved in microglial activation, inflammatory factor release, and oxidative stress, and activated microglia *via* the PLC/PKC/MAPK/NF-κB signaling cascade, providing a novel theoretical basis and therapeutic target for the inflammatory pathological mechanism of AD.

## Experimental procedures

### Animals

APP/PS1 double transgenic mice (B6. Cg-Tg (APPswe, PSEN1dE9) 85Dbo/Mmjax) were purchased from Changzhou Cavens Experimental Animal Co., Ltd, and housed in a specific pathogen-free facility at 25 °C ± 2 °C and 50 to 60% relative humidity under a 12-h light/12-h dark cycle, with free access to food and water. After 7 days of acclimation, 5-month-old male APP/PS1 mice and age- and sex-matched C57BL/6 WT mice were selected for *in vivo* experiments. According to different experimental purposes, the mice were divided into two independent animal cohorts: cohort one was used to assess disease-related phenotypes in APP/PS1 mice (n = 9/group); cohort two was treated with different methods to verify the regulatory effect of mCRP on PLC signaling pathway *in vivo*, the mice were randomly divided into four groups: WT + vehicle group, APP/PS1 + vehicle group, APP/PS1 + mCRP group and APP/PS1 + mCRP + U-73122 group (n = 3/group). Vehicle-treated mice received intraperitoneal injection of sterile buffer. The mCRP group received mCRP (200 μg/kg body weight, i.p.). The PLC inhibitor U-73122 (1 mg/kg body weight, i.p.) was administered 30 min before mCRP injection. All treatments were given once daily for 14 consecutive days. At the end of the treatment period, mice were anesthetized with sodium pentobarbital (i.p.) and sacrificed. Hippocampal tissues were isolated from a subset of mice and immediately stored at −80 °C for subsequent experiments. Other mice were fixed by cardiac perfusion and then placed in 4% paraformaldehyde for preservation. All animal experiments were approved by the Biomedical Ethics Committee of Xizang Minzu University (approval number: 2021-08) and performed in accordance with the 3R principles and institutional animal welfare guidelines.

### Microglial culture and treatment

Primary mouse microglial cells were cultured according to a previously reported method with minor modifications (42). C57BL/6 suckling mice within 24 h after birth were used. The brains were disinfected and isolated under sterile conditions, digested with 0.25% trypsin at 37 °C for 10 min, and the digestion was terminated using DMEM medium supplemented with 10% fetal bovine serum . After filtration and centrifugation, the cells were seeded into culture flasks for glial cell culture. Following culture at 37 °C in 5% CO_2_ for 10 to 12 days, primary microglia were isolated by tapping the culture flasks according to a previously described method and resuspended in DMEM medium containing 10% fetal bovine serum. The cells were verified by IBA-1 antibody detection and then used for subsequent experiments. BV2 mouse microglial cells (Procell Life Science & Technology Co., Ltd, Wuhan, China; Cat. No. CL-0493; authenticated by STR profiling) were subcultured at a ratio of 1:2 to 1:4 under the same culture conditions (37 °C, 5% CO_2_). The culture medium was replaced with fresh medium every 2 to 3 days.

In subsequent experiments, cells were treated with CRP (5/10/20 μg/ml), mCRP (5/10/20 μg/ml), and Aβ_42_ ADDLs (0.5 μM) alone or in combination, and with/without the presence of PLC specific inhibitor U-73122 (10 μM). Total protein and RNA were extracted from the cell lysates for Western blot and RT-qPCR analyses, respectively. The list of major chemical reagents used in this study is provided in Table S2.

### Preparation of CRP, mCRP and Aβ_42_ ADDLs

Treatment of CRP: Native human CRP protein was purchased from Yashraj Biotechnology Ltd (Cat. No. FCRhp-13-01, Lot No. FCR0407, concentration 2.81 mg/ml, purity 99.29%), diluted in 20 mM Tris buffer (pH 8.0), aliquoted, and stored at 2 to 8 °C for subsequent studies.

Preparation and quality testing of mCRP: CRP was dissolved in 8 M urea containing 5 mM EDTA (pH 7.4) to a final concentration of 200 μg/ml and incubated at 37 °C for 2 h. The resulting mCRP was purified by dialysis against sterile TBS (pH 7.4) for 48 h, aliquoted, and stored at −20 °C. Purity was verified by SDS-PAGE using graded protein loading (0.8, 1.6, and 3.2 μg per lane); a single band at approximately 23 kDa was detected, with no visible contaminating bands. Endotoxin contamination during mCRP preparation was excluded by polymyxin B neutralization assays as described previously (43, 44).

Preparation of Aβ_42_ ADDLs: Aβ_42_ monomers were prepared according to a previously reported protocol, diluted in DMEM, and incubated at 4 °C for 48 h (45).

### Clinical sample information

Patient Selection for AD: 65 patients with clinically diagnosed AD were recruited from the Department of Neurology at Chenzhou First People's Hospital, between March 2025 and February 2026. The cohort comprised 33 men and 32 women, ranging in age from 52 to 85 years. All participants underwent comprehensive clinical evaluation by experienced neurologists, including assessment of clinical symptoms, disease course, neuroimaging findings, and relevant laboratory indicators. AD was diagnosed according to the Chinese Guidelines for the Diagnosis and Treatment of AD and the diagnostic criteria established by the National Institute on Aging–Alzheimer’s Association (NIA–AA). Patients with cognitive impairment attributable to cerebrovascular disease, dementia with Lewy bodies, frontotemporal dementia, normal-pressure hydrocephalus, hypothyroidism, vitamin B deficiency, drug intoxication, brain tumors, or encephalitis were excluded. Thirty cognitively healthy individuals were recruited from the Health Management Center of the same hospital. Individuals with cardiovascular or inflammatory conditions, or other chronic diseases associated with elevated CRP levels, were excluded from the control group. All participants provided written informed consent for blood collection. The study was approved by the Ethics Committee of Chenzhou First People’s Hospital (Approval No. 2022028) and was conducted in accordance with the Declaration of Helsinki.

Peripheral venous blood samples were collected from all subjects under fasting conditions. Blood samples were centrifuged at 3000 rpm for 10 to 15 min to separate serum, which was stored at −80 °C until use to avoid repeated freezing and thawing. The serum total CRP concentrations were measured using a high-sensitivity immunoassay on a BN II automated specific protein analyzer (Siemens Healthcare Diagnostics), in accordance with the manufacturer’s instructions. Serum mCRP concentrations were determined using a modified double-antibody sandwich ELISA. The brief procedures were as follows: Anti-human C-reactive protein monoclonal antibody (purchased from MyBioSource; Cat. No. C1688; Lot No. 0000310540) was coated onto microplates and incubated overnight at 4 °C. After blocking, serum samples were added and incubated at 37 °C. Goat anti-human CRP polyclonal antibody (purchased from MyBioSource; Cat. No. MBS223280; Lot No. 155010) and horseradish peroxidase-labeled secondary antibody (purchased from Sigma-Aldrich; Cat. No. SLCJ3300; Lot No. 1003232204) were then added successively for reaction. Chromogenic reaction was developed using tetramethylbenzidine and terminated with 1 mol/L sulfuric acid. The absorbance was measured at a wavelength of 450 nm using a microplate reader, with correction at a reference wavelength of 630 nm. Routine serum biochemical parameters, including alanine aminotransferase, aspartate aminotransferase, total cholesterol, triglyceride, high density lipoprotein cholesterol, low density lipoprotein cholesterol, and apolipoprotein B/apolipoprotein A1ratio were measured using an ADVIA 2400 automated biochemical analyzer (Siemens Healthcare Diagnostics) and the corresponding commercial reagents, according to the manufacturer’s instructions.

### Transcriptomic data analysis based on the GEO public database

Considering the heterogeneity of different GEO datasets in terms of sample source, brain region composition and experimental design, this study did not directly merge the expression matrices of different datasets. The independent dataset analysis and validation strategy was used. GSE5281 was used for CRP-related expression pattern analysis, co-expression analysis and pathway enrichment analysis. GSE48350 was used to independently verify the changes in CRP expression in different brain regions. The GSE5281 dataset included brain tissue samples from AD patients (GSM238799-GSM238808) and matched healthy controls (GSM119628-GSM119637), with 10 samples in each group. All samples were detected by Affymetrix Human Genome U133 Plus 2.0 Array platform (GPL570). R software (version 4.0 or above; https://www.r-project.org) was used to generate gene expression heat maps and analyze the overall expression patterns of CRP and well-established classic AD-related genes, gene expression values were subjected to Z-score normalization across samples, and the CRP gene was highlighted and labeled prominently. Spearman correlation analysis was performed to screen genes at the genome-wide level, the expression correlation coefficients between CRP and each individual gene were systematically calculated, and a set of genes significantly positively correlated with CRP was identified (filtering criteria: correlation coefficient r > 0.3 and *p* < 0.05) for KEGG signaling pathway enrichment analysis. Based on the above results, this study further analyzed the expression correlation between CRP and genes associated with the PLC pathway.

### Immunofluorescence assay

After deep anesthesia, mice were sequentially perfused transcardially with precooled PBS and 4% paraformaldehyde. After fixation at 4 °C for 12 h, brain tissues were completely sunk in 10%, 20%, and 30% sucrose solutions in sequence, embedded, and sectioned at 20 to 30 μm using a cryostat. For immunofluorescence staining, brain tissues or cells were fixed with 4% paraformaldehyde at room temperature for 30 min, incubated with primary antibodies at 4 °C overnight, and then incubated with corresponding secondary antibodies after washing. Nuclei were counterstained with DAPI. After coverslipping, images were captured using a laser confocal microscope (Leica TCS SP8). Fluorescence intensity quantification, nuclear/cytoplasmic ratio calculation, and colocalization analysis of related molecules were performed using ImageJ software (https://imagej.net/ij/). The quantitative method at the single-cell level was used in this study, to exclude the impact of inconsistent cell density. Individual microglia was identified by labeling the nucleus with DAPI combined with cell morphology, and fluorescence intensity was analyzed for each cell area. The antibody information used in this study is shown in Table S3.

### Western blot

Tissue or cell samples were lysed with RIPA lysis buffer containing protease and phosphatase inhibitors (Beyotime, P0013B; Roche, 04693132001). After separation by SDS-PAGE, proteins were transferred onto PVDF membranes. Following blocking, membranes were incubated with primary antibodies against CRP (1:5000), IL-6 (1:5000), IL-1β (1:2000), TNF-α (1:2000), PKC (1:5000), ERK1/2 (1:2000), p-ERK1/2 (1:5000), JNK (1:2000), p-JNK (1:5000), p38 (1:5000), p-p38 (1:5000), NF-κB p65 (1:5000), and p-NF-κB p65 (1:5000). After washing with 1 × TBS, membranes were incubated with HRP-conjugated secondary antibodies at room temperature for 2 h. Finally, immunoreactive bands were visualized using ECL luminescent reagent and a Western blot imaging system (Bio-Rad). The gray value quantitative analysis was performed with reference to internal reference proteins. The antibody information used in this study is shown in Table S3.

### Total RNA extraction and RT-qPCR analysis

Hippocampal tissue or cell samples were collected and lysed with TRIGene lysis buffer, followed by grinding at low temperature. After liquid phase separation, RNA was precipitated and collected using isopropanol, dissolved in an appropriate volume of nuclease-free water by repeated pipetting, and then assessed for concentration, purity and integrity using a Nanodrop spectrophotometer and RNA electrophoresis. Qualified RNA samples were subjected to reverse transcription according to the instructions of the reverse transcription kit (Thermo Fisher Scientific, M1631) to obtain cDNA templates for each group. Subsequently, RT-qPCR was performed following the manufacturer’s protocol for 2 × Real Star Fast SYBR qPCR Mix, and the relative mRNA expression levels of each target gene were calculated using the^△△^ Ct method with an RT-qPCR instrument. The primer sequences used in this study are listed in Table S4.

### Detection of mitochondrial membrane potential (ΔΨm) and ROS

Primary microglia was directly detected using a JC-1 kit. The fluorescence intensities of red (aggregated form) and green (monomeric form) signals were quantified to evaluate changes in mitochondrial membrane potential (ΔΨm) in primary microglia after treatment with various concentrations of CRP or mCRP for 12 h. The intracellular ROS content in primary microglia was assessed using a DCFH-DA detection kit. Briefly, cells were seeded at a density of 1.0 × 10^6^ to 2.0 × 10^7^, and the DCFH-DA probe was diluted in serum-free medium to a final concentration of 10 μM. After incubation at 37 °C for 30 min and washing with PBS, cells were observed directly under a laser confocal microscope, and the mean fluorescence intensity was quantified using Image J software.

### Flow cytometry

Primary microglia was coincubated with different concentrations of CRP or mCRP, respectively. After 12 h, cell samples were collected and incubated with CD86 antibody (1:300) at 4 °C for 30 min in the dark. After washing with PBS and centrifugation, the cell suspension was detected using a Beckman flow cytometer, and data were analyzed and processed with Kaluza 2.1 software (https://www.mybeckman.cn/flow-cytometry/software/kaluza).

### Statistical analysis

To reduce observer bias, it is declared that all analyses in this study were conducted using blinding methods, and researchers were unaware of grouping or experimental conditions during the raw data collection and recording process. All measurement data were presented as the mean ± SD. All statistical analyses were performed using GraphPad Prism 10.1.2 software (https://www.graphpad.com/scientific-software/prism/). For comparisons between two independent groups, the independent-sample Student’s *t* test was used for data with normal distribution and homogeneity of variance, while the Mann-Whitney U nonparametric rank-sum test (Wilcoxon rank-sum test) was applied for data that did not conform to a normal distribution, as well as for serum markers and gene expression data. One-way analysis of variance (one-way ANOVA) followed by the LSD *post hoc* multiple comparisons test was used for comparisons among multiple groups with a single factor. Two-way analysis of variance (two-way ANOVA) followed by the Tukey *post hoc* multiple comparisons test was used for comparisons involving two independent influencing factors. A *p* value of less than 0.05 was considered statistically significant (ns, no significant difference), with significance levels defined as ∗*p* < 0.05, ∗∗*p* < 0.01, ∗∗∗*p* < 0.001 and ∗∗∗∗*p* < 0.0001.

## Data availability

The data associated with the paper are not publicly available but are available from the corresponding author (Qian Chen) on reasonable request.

## Supporting information

This article contains supporting information.

## Conflict of interest

The authors declare that they have no conflicts of interest with the contents of this article.
