# Supplementary material for: Monomeric C-reactive protein potentiates microglia-mediated neuroinflammation in Alzheimer’s disease via the PLC-PKC-MAPK-NF-κB pathway
Source: J Biol Chem. 2026 Sep 8;302(10):113527. doi: 10.1016/j.jbc.2026.113527 (PMC13671087; doi:10.1016/j.jbc.2026.113527)
Supplement: Supplementary file 1 [file mmc1.docx]

**Supplementary Materials**

**Supplementary Tables:**

**Supplementary Table 1.Clinical characteristics of study participants.Data are presented as n (%) for categorical variables and median (interquartile range, IQR) for continuous variables.**

| **Characteristic** | **AD (n=65)** | **Control (n=30)** | **P value** |
| --- | --- | --- | --- |
| Sex, n (%) |  | | 0.880 |
| Male | 33 | 14 |  |
| Female | 32 | 16 |  |
| Age (years) | 69 (62–75) | 60 (52–68) | <0.001 |
| BMI (kg/m²) | 25.4 (23.0–27.6) | 24.0 (21.0–26.8) | 0.067 |
| AST (U/L) | 21 (14–27) | 23 (18–29) | 0.257 |
| ALT (U/L) | 11 (7–14) | 21 (14–28) | <0.001 |
| TC (mmol/L) | 3.9 (3.1–4.5) | 5.5 (4.8–6.2) | <0.001 |
| TG (mmol/L) | 2.7 (1.5–3.5) | 1.9 (1.0–2.5) | 0.045 |
| ApoA1 (g/L) | 1.3 (1.0–1.5) | 2.0 (1.6–2.3) | <0.001 |
| ApoB (g/L) | 0.8 (0.6–0.9) | 1.4 (1.1–1.6) | <0.001 |
| HDL (mmol/L) | 1.0 (0.7–1.2) | 1.5 (1.2–1.8) | <0.001 |
| LDL (mmol/L) | 3.4 (2.4–4.3) | 3.3 (2.6–4.0) | 0.983 |
| CRP (mg/L) | 7.5 (1.5–12.3) | 1.3 (0.8–2.1) | 0.177 |
| mCRP (ng/mL) | 0.03 (0.00–0.05) | 0.01 (0.00–0.03) | 0.048 |

The normality of continuous variables was assessed using the Shapiro–Wilk test. Because all continuous variables showed non-normal distributions, comparisons between groups were performed using the Mann–Whitney U test. Differences in sex distribution were analyzed using Pearson’s χ² test. A two-sided P value < 0.05 was considered statistically significant.

**Abbreviations:** AD, Alzheimer’s disease; BMI, body mass index; AST, aspartate aminotransferase; ALT, alanine aminotransferase; TC, total cholesterol; TG, triglycerides; ApoA1, apolipoprotein A1; ApoB, apolipoprotein B; HDL-C, high-density lipoprotein cholesterol; LDL-C, low-density lipoprotein cholesterol; CRP, C-reactive protein; mCRP, monomeric C-reactive protein; IQR, interquartile range.

**Supplementary Table 2.Detailed information of reagents and antibodies used in this study.**

| **Category** | **Reagent/Antibody** | **Application** | **Catalog No.** | **Manufacturer** |
| --- | --- | --- | --- | --- |
| Cell culture reagent | DMEM medium | Cell culture | C11995500BT | Gibco,USA |
| Cell culture reagent | 0.25% Trypsin–EDTA | Cell dissociation | 25200-072 | Gibco,USA |
| Cell culture reagent | Fetal bovine serum (FBS) | Cell culture supplement | C04001-500 | DASHI Biotech, Shanghai,China |
| Cell culture reagent | Penicillin–Streptomycinsolution | Antibiotic supplement | SV30010 | HyClone,USA |
| Cell culture reagent | Neurobasal™-A medium | Primary microglia culture | 10888022 | Gibco,USA |
| Cell culture reagent | B27® supplement | Primary cell culture | 17404-044 | Gibco,USA |
| Cell adhesion reagent | Poly-D-lysine hydrobromide | Cell coating | P0899-50MG | Sigma-Aldrich,USA |
| Fixation reagent | 4% Paraformaldehyde | IF fixation | MM1506 | Maokang Biotechnology,China |
| Permeabilization reagent | Triton™ X-100 | IF staining | S15022 | Maokang Biotechnology,China |
| Permeabilization reagent | Triton™ X-100 Surfact-Amps™ | IF staining | 85111 | Thermo Fisher Scientific,USA |
| Nuclear staining reagent | Hoechst 33342 | Nuclear staining | C1027 | Beyotime Biotechnology, China |
| Mounting reagent | DAPI-Fluoromount-G™ | Fluorescence mounting | 0100-20 | SouthernBiotech, USA |
| Flow cytometry antibody | PE-conjugated anti-CD86 antibody | Flow cytometry | 159204 | Bio-Rad Laboratories, USA |
| ROS detection reagent | ROS assay kit | ROS detection | S0033M | Beyotime Biotechnology, China |
| Mitochondrial ROS probe | MitoSOX™ Red | MitoROS detection | HY-D1055 | MedChemExpress,USA |
| Mitochondrial membrane potential assay | JC-1 kit | ΔΨm detection | M8650 | Solarbio Life Sciences,China |
| PLC inhibitor | U-73122 | PLC inhibition | HY-13419/CM05929 | MedChemExpress / Proteintech |
| Endotoxin neutralization reagent | Polymyxin B | LPS neutralization assay | HY-149179 | MedChemExpress,USA |
| Reverse transcription reagent | StarScript III RT Premix with gDNA removal | RT-qPCR | A230 | GenStar, China |
| RNA extraction reagent | TRIGene™ reagent | RNA extraction | P118-05 | GenStar, China |
| qPCR reagent | 2× RealStar Fast SYBR qPCR Mix | RT-qPCR | A301-5 | GenStar, China |

**Supplementary Table 3.Primary antibodies and secondary antibodies used in this study.**

| **Target** | **Host/Type** | **Application** | **Catalog No.** | **Manufacturer** |
| --- | --- | --- | --- | --- |
| pan-PKC | Rabbit polyclonal | WB | 12919-1-AP | Proteintech, USA |
| p-JNK (Tyr185) | Rabbit monoclonal | WB | 80024-1-RR | Proteintech, USA |
| JNK | Rabbit monoclonal | WB | P22712 | ProMab, USA |
| p-ERK1/2 (Thr202/Tyr204) | Rabbit monoclonal | WB | 80031-1-RR | Proteintech, USA |
| ERK1/2 | Rabbit polyclonal | WB | T40071 | Abmart, China |
| p-p38 MAPK (Thr180/Tyr182) | Rabbit polyclonal | WB | 28796-1-AP | Proteintech, USA |
| p38 MAPK | Rabbit polyclonal | WB | 14064-1-AP | Proteintech, USA |
| NF-κB p65 | Rabbit polyclonal | WB/IF | 10745-1-AP | Proteintech, USA |
| Phospho-NF-κB p65 (Ser536) | Rabbit monoclonal | WB | 80379-2-RR | Proteintech, China |
| NF-κB p65 | Rabbit monoclonal | WB | PT0352R | Immunoway, China |
| Phospho-NF-κB p65 (Ser536) | Rabbit monoclonal | WB | PT0633R | Immunoway, China |
| CRP | Mouse monoclonal | WB/IF | 66250-1-Ig | Proteintech, USA |
| IL-6 | Rabbit polyclonal | WB | 21865-1-AP | Proteintech, USA |
| IL-1β | Rabbit polyclonal | WB | 26048-1-AP | Proteintech, China |
| TNF-α | Mouse monoclonal | WB | 60291-1-Ig | Proteintech, USA |
| β-Actin | Mouse monoclonal | WB | P30002M | Abmart, China |
| Iba1 | Rabbit polyclonal | IF | 019-19741 | FUJIFILM Wako, Japan |
| Goat anti-rabbit IgG (H+L) | Goat polyclonal | IF | ab150084 | Abcam, UK |
| Donkey anti-rabbit IgG (H+L) | Donkey polyclonal | IF | ab150067 | Abcam, UK |
| Goat anti-mouse IgG (H+L) | Goat polyclonal | IF | ab150113 | Abcam, UK |

**Supplementary Table 4.Primer sequences used for RT-qPCR.**

| **Gene** | **Forward primer (5′–3′)** | **Reverse primer (5′–3′)** |
| --- | --- | --- |
| Mus-Tnfα | CAGCGCTGAGGTCAATCTGCC | TGCCCGGACTCCGCAA |
| Mus-Il6 | CTGCAAGAGACTTCCATCCAG | AGTGGTATAGACAGGTCTGTTGG |
| Mus-IL1β | GAAATGCCACCTTTTGACAGTG | TGGATGCTCTCATCAGGACAG |
| Mus-CD86 | TGTTTCCGTGGAGACGCAAG | TTGAGCCTTTGTAAATGGGCA |
| Mus-Mrc1 | CTCTGTTCAGCTATTGGACGC | CGGAATTTCTGGGATTCAGCTTC |
| Mus-CRP | AGTCTGCACAAGGGCTACAC | AAGTCACCGCCATACGAGTC |
| Mus-β-actin | GGCTGTATTCCCCTCCATCG | CCAGTTGGTAACAATGCCATGT |

**Supplementary Figures:**

**Supplementary Figure 1.Spearman correlation analysis between circulating mCRP levels and clinical biochemical parameters.** Scatter plots show the associations between serum mCRP concentrations and BMI, AST, ALT, TC, TG, ApoB/ApoA1, HDL, LDL, and total CRP concentrations. Spearman rank correlation analysis was performed to determine the correlation coefficients (r) and corresponding P values. Red lines indicate the fitted trend lines, with shaded areas representing the corresponding 95% confidence intervals. Serum mCRP concentrations were significantly negatively correlated with TC (r = −0.244, P = 0.017) and positively correlated with total CRP concentrations (r = 0.231, P = 0.024). No statistically significant correlations were observed between mCRP and BMI, AST, ALT, TG, ApoB/ApoA1, HDL, or LDL (all P > 0.05).

**Supplementary Figure 2. Forest plot of multivariate linear regression analysis identifying independent clinical correlates of circulating mCRP levels.** Multivariable linear regression analysis was performed with circulating plasma mCRP concentration as the dependent variable. The model was adjusted for diagnostic group (AD versus control), age, sex, BMI, hepatic biochemical parameters (AST and ALT), and lipid-related parameters, including TC, TG, HDL, LDL, and the ApoB/ApoA1 ratio. The forest plot displays the adjusted regression coefficients (β) and corresponding 95% confidence intervals (CIs) for each covariate. Each point represents the estimated β coefficient, and horizontal bars indicate the 95% CI. The vertical dashed line represents the null value (β = 0). Associations were considered statistically significant when the 95% CI did not cross zero.

**Supplementary Figure 3. Sex-stratified comparison of circulating mCRP levels in healthy controls and patients with Alzheimer’s disease.** Participants were stratified into four groups according to clinical diagnosis and sex: male healthy controls, female healthy controls, male patients with Alzheimer’s disease (AD), and female patients with AD. Open circles represent individual serum mCRP concentrations. Sex differences in serum mCRP levels were assessed separately within the healthy control group and the AD group using the Mann–Whitney U test. No significant difference was observed between male and female healthy controls (P = 0.205), or between male and female patients with AD (P = 0.904). All individual data points are shown.

**Supplementary Figure 4. CRP and mCRP enhance Aβ_42_-ADDLs-induced inflammatory responses in BV-2 microglial cells.** BV-2 microglial cells were treated with six experimental conditions: control, CRP (5 μg/mL), mCRP (5 μg/mL), Aβ_42_-ADDLs (0.5 μM), CRP + Aβ_42_-ADDLs, and mCRP + Aβ_42_-ADDLs.(A) Representative western blot images showing the protein expression levels of pro-inflammatory cytokines IL-6, IL-1β, and TNF-α. β-Actin was used as the loading control.(B–D) Quantification of IL-6, IL-1β, and TNF-α protein expression levels.(E–G) Relative mRNA expression levels of IL-6, IL-1β, and TNF-α determined by quantitative real-time PCR.Individual data points represent independent biological replicates. Data are presented as mean ± standard deviation (SD). Statistical analysis was performed using one-way ANOVA followed by Tukey’s multiple comparisons test. ns, not significant; *P < 0.05, **P < 0.01, ***P < 0.001, ****P < 0.0001.

**Supplementary Figure 5. Effect of gradient concentrations of mCRP on the viability of primary mouse microglia determined by CCK-8 assay.** Primary mouse microglia were treated with increasing concentrations of mCRP, and cell viability was evaluated using the CCK-8 assay. Cell viability was normalized to the untreated control group and expressed as a percentage of control values.mCRP treatment at concentrations of 5–20 μg/mL did not significantly affect microglial cell viability, whereas higher concentrations (≥40 μg/mL) resulted in a significant reduction in cell viability. Data are presented as mean ± standard deviation (SD) from five independent biological replicates (n = 5). Statistical analysis was performed using one-way ANOVA followed by Tukey’s multiple comparisons test. ns, not significant; ***P < 0.001; ****P < 0.0001.

**Supplementary Figure 6.Characterization of prepared monomeric C-reactive protein (mCRP) by non-reducing SDS-PAGE and immunoblotting.** Increasing amounts of prepared mCRP (0.8, 1.6, and 3.2 μg) were subjected to non-reducing SDS-PAGE followed by immunoblot analysis. A single immunoreactive band corresponding to the expected molecular weight of monomeric CRP (~23 kDa) was detected, with increased signal intensity corresponding to the loaded protein amounts. No apparent higher molecular weight CRP species or additional nonspecific protein bands were observed, indicating the high quality and consistency of the prepared mCRP samples used for subsequent experiments.

**Supplementary Figure 7.Effect of polymyxin B treatment on mCRP-induced inflammatory gene expression in primary mouse microglia.** (A-C) Primary mouse microglia were divided into six groups: control, polymyxin B (PMB) alone, lipopolysaccharide (LPS), LPS + PMB, mCRP, and mCRP + PMB groups. Cells were treated for 12 h, and the mRNA expression levels of IL-6, Tnfα, and IL-1β were determined by reverse transcription quantitative PCR (RT-qPCR). Gene expression levels were normalized to the corresponding housekeeping gene expression levels. PMB treatment significantly attenuated LPS-induced upregulation of inflammatory genes, whereas PMB neutralization did not significantly affect mCRP-induced increases in Il6, Tnfα, and Il1β expression. These results indicate that the pro-inflammatory activity of the prepared mCRP was primarily attributable to the intrinsic biological activity of mCRP rather than potential endotoxin contamination.Data are presented as mean ± SD. Statistical analysis was performed using one-way ANOVA followed by Tukey’s multiple comparison test. ns, not significant; ns, not significant; *P < 0.05, **P < 0.01, ****P < 0.0001.
